# Diagnostic accuracy of the point-of-care standard G6PD test™ (SD Biosensor) for glucose-6-phosphate dehydrogenase deficiency: a systematic review and meta-analysis

**DOI:** 10.1186/s12936-024-05144-1

**Published:** 2024-11-02

**Authors:** Juan Camilo Martínez, Viviana Vélez-Marín, Mary Lopez-Perez, Daniel F. Patiño-Lugo, Ivan D. Florez

**Affiliations:** 1 Unit of Evidence and Deliberation for Decision Making UNED, Medical Research Institute, School of Medicine, University of Antioquia, Medellin, Colombia; 2 Centre for Medical Parasitology, Department for Immunology and Microbiology, Faculty of Health and Medical Sciences, University of Copenhagen, Copenhagen, Denmark; 3 Department of Pediatrics, School of Medicine, University of Antioquia, Av. St 70 No. 52-21, Medellin, Colombia; 4 School of Rehabilitation Science, McMaster University, Hamilton, Canada; 5 Pediatric Intensive Care Unit, Clinica Las Americas-AUNA, Medellin, Colombia

**Keywords:** Systematic review, Metanalysis, Glucose-6-phosphate dehydrogenase, Malaria, Point-of-care test, Standard G6PD™

## Abstract

**Background:**

Glucose-6-Phosphate Dehydrogenase deficiency (G6PDd) is a common genetic enzymopathy that can induce haemolysis triggered by various factors, including some anti-malarial drugs. Although many Point-of-Care (PoC) tests, such as Standard G6PD™ are available to detect G6PDd, its pooled diagnostic test accuracy (DTA) remains unknown.

**Methods:**

To estimate the DTA of StandG6PD-BS at various thresholds of G6PDd, a systematic review with a DTA meta-analysis were conducted, searching EMBASE, MEDLINE, and SciELO databases up to April 4, 2024.The included studies were those that measured G6PD activity using StandG6PD-BS (reference test) and spectrophotometry (gold standard) in patients suspected of having G6PDd. The risk of bias (RoB) of the studies was assessed using the QUADAS-2 tool and the certainty of evidence (CoE) with the GRADE approach. For the estimation of within-study DTA, a random-effect bivariate meta-analysis was performed to determine the pooled sensitivity and specificity for 30%, 70%, and 80% enzyme levels’ thresholds, and a graphical analysis of the heterogeneity using crosshair and Confidence Regions on receiver operating characteristic (ROC) space plots.

**Results:**

After screening 2496 reports, four studies were included with 7864 participants covering all thresholds. Two studies had high RoB in QUADAS-2 domains 2 and 3, and the others had low RoB, with low, moderate, and high heterogeneity at the 30%, 70%, and 80% thresholds, respectively. The pooled sensitivity was 99.1%, 95.7%, and 90% for 30%, 70%, and 80% thresholds, respectively. The pooled specificity was 97.4%; 92.9%; and 89.0% for 30%, 70%, and 80% thresholds, respectively.

**Conclusion:**

StandG6PD-BS is a PoC test with high sensitivity and specificity to detect G6PDd at different thresholds.

**Supplementary Information:**

The online version contains supplementary material available at 10.1186/s12936-024-05144-1.

## Background

Glucose-6-phosphate dehydrogenase deficiency (G6PDd) is a genetic disorder linked to the X chromosome, with hemizygous males and homozygous females having a deficient activity phenotype (< 30% of enzyme levels). In contrast, heterozygous females may have a normal (> 80%) or intermediate activity phenotype (30% to 80% enzyme level) [1]. Individuals with deficient activity may experience haemolytic episodes triggered by intrinsic or extrinsic factors such as medications or certain foods [2]. G6PDd is the most common enzymopathy in humans, with variable frequencies and distinctive region-specific distribution [3]. It is particularly common in malaria-endemic regions [1, 4], with an estimated frequency of 8–10% (~ 350 to 400 million cases per year) and over 200 identified genetic polymorphisms [5]. This overlapping is attributed to the protective effect of G6PDd against malaria [1].

Malaria caused by *Plasmodium vivax* is geographically widespread and counts for most cases outside Africa, particularly in the Americas and South-East Asia [6]. Primaquine (PQ) and the novel tafenoquine (TQ) are the only two approved drugs for treating hepatic stages and are used for the radical cure of uncomplicated malaria by *P. vivax* [5, 7]. However, these drugs may precipitate haemolytic crises in individuals with G6PDd [1, 5, 7], with the severity of the reaction being proportional to the dose of the medication received and the enzyme genotype [5, 7]. To ensure safe administration of these drugs, the World Health Organization (WHO) recommends testing for G6PDd in those requiring treatment with PQ [7]. This could be achieved by using Point-of-Care (PoC) tests to determine G6PD activity before administering PQ and TQ, as has been recommended by multiple studies [8].

Several PoC tests are currently available to determine the G6PD activity, but they have different performance regarding sensitivity and specificity, mainly due to the kind of test and blood source [9, 10]. Since the gold standard for G6PD measurement (spectrophotometry) is not suitable for PoC testing, as requires advanced laboratory infrastructure and skilled personnel, qualitative tests have been developed with variable diagnostic performance and operational characteristics [11–13]. While those tests discriminate between normal and deficient G6PD activity and, therefore, are sufficient to guide PQ treatment, they may not be dependable enough to prevent drug-induced haemolysis with the introduction of TQ. Consequently, more reliable diagnostic tests are required, and one of such is the semi-quantitative assay Standard G6PD™ (SD Biosensor, Republic of Korea), an enzymatic colourimetric assay intended to aid the detection of G6PDd [14]. This PoC test provides a numeric measurement of G6PD enzymatic activity and total haemoglobin (Hb) concentration in fresh capillary and venous human whole blood specimens [14] and allows classification of the G6PD activity as deficient, intermediate, or normal according to thresholds provided by the manufacturer [14].

Despite the recommendation for PoC quantitative or semi-quantitative testing before administering anti-malarial treatment, there is only one synthesis of the diagnostic test accuracy (DTA) of the Standard G6PD™ test manufactured by SD Biosensor (StandG6PD-BS) [15] in which the authors pooled the individual results of various studies without a complete systematic review approach, risk of bias, and certainty of evidence assessment. In this systematic review and metanalysis, the pooled DTA of the StandG6PD-BS for different thresholds of G6PD activity was estimated.

## Methods

### Protocol and registration

This systematic review and DTA meta-analysis were conducted and reported following the Preferred Reporting Items for Systematic Reviews and Meta-analyses of DTA Studies (PRISMA-DTA) guidelines and the Cochrane Handbook for Systematic Reviews of Diagnostic Test Accuracy [16]. The protocol was registered in the PROSPERO database (CRD42022311085).

### Search and study selection

Two authors (JCM, VVM) performed a structured search in MEDLINE (via PubMed), EMBASE, and SciELO databases on August 2nd, 2022, and updated it on January 31, 2023, on June 30, 2023, and on April 4, 2024, without language or date restrictions. The search strategy is outlined in Table S1. Cross-sectional prospective or retrospective studies that measured G6PD activity levels using the reference standard enzymatic test (spectrophotometry G6PD assay) and the reference test (StandG6PD-BS) were included, regardless of the population where those were carried out. The studies must have reported enough data to calculate diagnostic performance measures, i.e., true positives (TP), true negatives (TN), false positives (FP), and false negatives (FN), for at least one threshold (30%, 70%, and 80%) of G6PD activity.

Using Rayyan software (Rayyan QCRI, Qatar) [17], two authors screened titles and abstracts retrieved from searches, and only those records considered eligible by both reviewers were retrieved in full texts for the next stage. The same authors (JCM, VVM) reviewed the potentially eligible full texts, independently and in duplicate, based on the pre-specified inclusion criteria. Those studies considered eligible by both reviewers were included. Disagreements were resolved by consensus among the reviewers and with the participation of a third reviewer, if needed (MLP, IDF).

The same authors (JCM, VVM) independently and in duplicate extracted the data from the included studies using a prespecified data extraction form designed in Google Forms (Google LLC, US), which was discussed and piloted among the research team. For each study, the following information was extracted: first author, year of publication, title, population, data for every threshold recommended by the manufacturer (SD Biosensor), number of participants, age (mean and standard deviation), sex, type of blood sample (venous or capillary), and the data needed for 2 × 2 contingency tables (TP, FP, FN, and TN). Disagreements in the data extraction process were discussed between the reviewers.

### Risk of bias assessment and certainty of the evidence

Two authors (JCM, VVM) independently assessed the Risk of Bias (RoB) for the included studies using the Quality Assessment of Diagnostic Accuracy Studies 2 (QUADAS-2) tool [18]. The QUADAS-2 instrument evaluates the RoB of DTA studies with four domains (patient selection, index test, reference standard, and flow and timing), judging each as high, low, or unclear in risk of bias and concerns regarding applicability. The authors discussed any disagreements and resolved them through consensus, since the agreement was achieved in all the cases, there was no need for involvement of a third reviewer.

The Certainty of the Evidence (CoE) was assessed using the GRADE framework for DTA systematic reviews [19, 20]. This approach evaluates four criteria: RoB (judged using the QUADAS-2 assessment), indirectness (with the evaluation of the study to our research question), inconsistency (with a visual inspection of the crosshair plots), publication bias (with funnel plot if feasible) and imprecision (with the width of the confidence interval), dose–response (with the changes of the sensitivity and specificity in different thresholds), and rates the certainty of the evidence in high, moderate, low, and very low.

### Statistical analysis

R Software and the package *mada* (version 4.1.2, The R Foundation for Statistical Computing) for the statistical analyses [21] and the GRADE Pro GDT platform (McMaster University 2015, developed by EvidencePrime, Inc) for creating the Summary of Findings (SoF) tables were used. First, we calculated sensitivity, specificity, positive likelihood ratio (+ LR), negative likelihood ratio (-LR), and diagnostic odds ratio (DOR) at every threshold. The cut-off recommended for the StandG6PD-BS test manufacturer is a valuable tool for therapeutic decisions because enzyme levels define the use of specific treatment. Thus, enzymatic levels above 30% allow using PQ and other drugs at specific doses; levels above 70% allow using TQ and any PQ treatment schedule; and levels above 80% define a normal G6PD activity. Then, the type of blood sample data at each threshold with the *Reistma* model was fitted [22], a bivariate random-effect meta-analysis that can model the heterogeneity found in the included studies. With this approach, the pooled sensitivity, specificity, + LR, and –LR and their corresponding 95% confidence intervals (95% CI) utilizing model estimations was calculated, using data only from female participants at 70% and 80% thresholds. The results for venous blood samples were pooled because the combined capillary samples were too small and not measured in all the studies.

For between-studies heterogeneity, visual inspection of forest plots for DTA pooled measures, crosshair plots (sensitivity vs. false positive rate), confidence regions in the *Receiver Operating Characteristic (*ROC) space, evaluating the overlap in the confidence intervals, and *chi-square* test for homogeneity (*p-*values < 0.05 were considered significant, and therefore as with heterogeneity) was used. There were not enough studies to fit the sensitivity and subgroup analysis models as stated in the registered protocol (sex and studies conducted in participants of African ethnicities).

## Results

### Search results

**Fig. 1:**
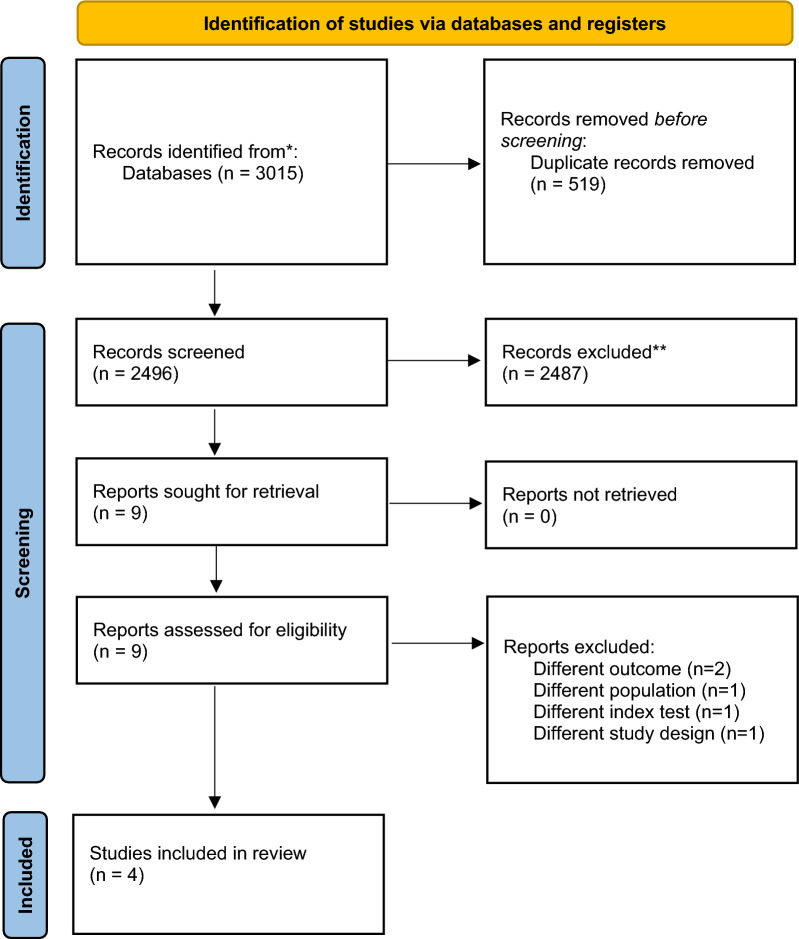
PRISMA flow diagram indicating the process of inclusion and exclusion of studies

3015 records and after removing duplicates were obtained, 2496 unique reports and identified nine potentially eligible studies were screened. After the full-text assessment, five studies were excluded and four studies were included that met the inclusion criteria for qualitative and quantitative synthesis (Fig. 1).

### Included and excluded studies

Five studies were excluded (Table S2) because of the different populations, reference tests, or outcomes (i.e., neither provides DTA measures nor data to calculate them; Table S2). Table 1 summarizes the characteristics of the four included studies [23–26]. These studies were conducted in the United States (U.S.) [23, 26], United Kingdom (U.K.) [23], Brazil [24], Bangladesh [25], and Thailand [26]. In total, they included 3122 (30% thresholds of the StandG6PD-BS), 2371 (70%), and 2371 (80%) participants for each test analysis, respectively. All studies were cross-sectional DTA, two included healthy adults (≥ 18 years old) [23, 26], one included participants older than 2 years [24], and three studies included individuals with known G6PD status [24–26], only one reported the ethnic background of the participants [23]. The reference test used in all the studies was spectrophotometry for the G6PD kit (Pointe Scientific).

**Table 1:** Characteristics of included studies

First Author	Year	Study type	Countries	Study context	Inclusion criteria	Sample type	Threshold	N for test operative characteristics (2 × 2 tables)					Reference (index) test	Gold Standard	Observation	Funding
								TP	FN	FP	TN	Total				
Pal [23]	2021	Cross-sectional DTA	U.S. (three clinical centers) U.K	Healthy volunteers blood donors in different clinical centers	Healthy adults, ≥ 18 years old They signed written informed consent	Venous	30%	56	0	26	708	790	STANDARD^™^ G6PD (SD Biosensor)	Spectrophotometry for G6PD using the kit Pointe Scientific	None	UK’s FCDO, Bill & Melinda Gates fdn
							70%	82	1	49	658	790				
							80%	85	16	58	631	790				
Zobrist [24]	2021	Cross-sectional DTA	Brazil	Febrile patients seeking care at the Manaus (924) and Porto Velho clinics (812)	Participants ≥ 2 years old plus 69 belonged through an enriched sample for G6PD known status	Venous	30%	56	0	23	1583	1662	STANDARD^™^ G6PD (SD Biosensor)	Spectrophotometry for G6PD using the kit Pointe Scientific	Measurement of temperature and humidity data at the time of testing that can affect PoC enzymatic activity test	UK’s FCDO, Bill & Melinda Gates fdn.,the National Institutes of Health
							70%	31	1	31	848	911				
							80%	41	20	21	829	911				
Alam [25]	2018	Cross-sectional DTA	Bangladesh	Participants were recruited in the Chittagong Hill Tracts districts (south-eastern)	Convenience sample from a cohort of adult with known G6PD status	Venous	30%	30	0	0	76	106	STANDARD^™^ G6PD (SD Biosensor)	Spectrophotometry for G6PD using the kit Pointe Scientific	Review of test results changes in blood samples stored at room temperature (24 to 26ºC) and 4ºC	Wellcome Trust, Australian DFAT, Bill & Melinda Gates fdn
							70%	48	3	8	47	106				
							80%	58	3	9	36	106				
Pal [26]	2018	Cross-sectional DTA	U.S	Healthy volunteers blood donors in two clinical centers (New York and Miami)	Healthy adults, ≥ 18 years old, with an African American origin	Fresh venous blood	30%	84	0	10	320	414	STANDARD^™^ G6PD (SD Biosensor)	Spectrophotometry for G6PD using the kit Pointe Scientific (for Thailand, the Trinity Biotech assay was used)	None	UK’s DFID, Bill & Melinda Gates fdn., MORU-Wellcome Trust
							70%	105	5	9	295	414				
							80%	115	6	40	253	414				
		Cross-sectional DTA	Thailand	Participants attended two clinical centers located north and south of Mae Sot that serve a migrant population of Burman and Karen ethnic groups	Adults with known G6PD status joined the study to achieve a convenience sample	Frozen venous blood	30%	54	0	5	91	150				
							70%	96	5	9	40	150				
							80%	103	4	11	32	150				

*TP* True Positives, *FN* False Negatives, *FP* False Positives, *TN* True Negatives, *G6PD* Glucose-6-Phosphate Dehydrogenase, *DTA* Diagnostic Test Accuracy, *U.S.* United States of America, *U.K.* United Kingdom, *FCDO* Foreign, Commonwealth & Development Office, *fdn.* Foundation, *DFAT* Department of Foreign Affairs and Trade, *DFID* Department for International Development, *MORU* Mahidol Oxford Tropical Medicine Research Unit

### Risk of bias (RoB)

**Fig. 2:**
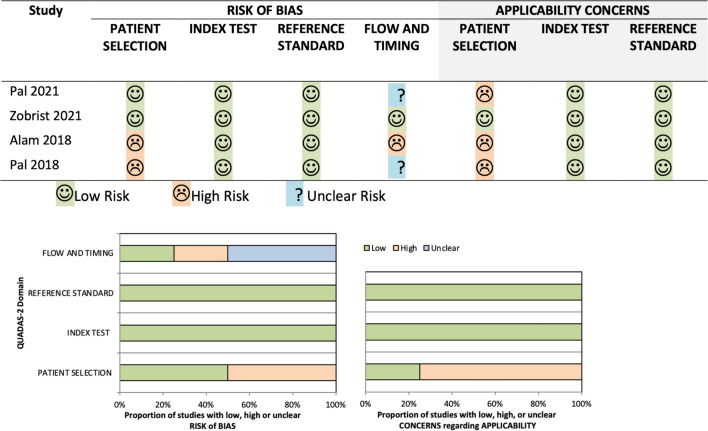
RoB summary judgements about each included study using QUADAS-2 tool

Two studies were judged as high [25, 26], and two as low RoB in domain one [23, 24] (patient selection) of the QUADAS-2 tool. One study was judged high [25], two unclear [23, 26], and one low RoB in domain four [24] (flow and timing). All studies had low RoB and applicability concerns in domains two and three (reference test and standard). This information is presented as a table and diagram using the QUADAS-2 tool resources (Fig. 2).

### Diagnostic test accuracy from primary studies and pooled data

**Fig. 3:**
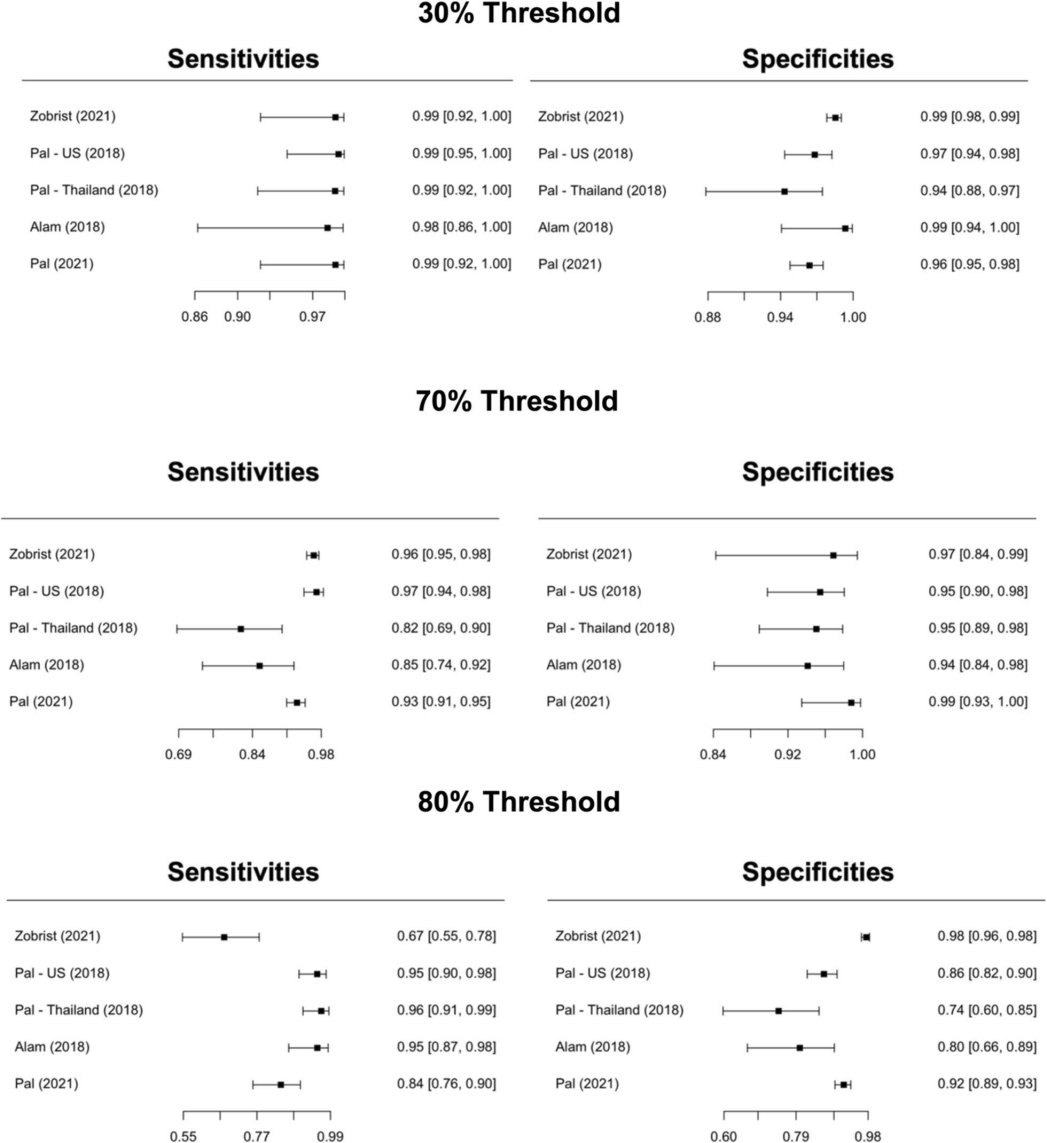
Pooled Sensitivity and Specificity for Standard^™^ G6PD (SD Biosensor) in venous blood samples

Sensitivities and specificities from primary studies are presented in Fig. 3. Sensitivity ranged from 91 to 99%, whereas specificities were from 89 to 97% for 30% and 80% thresholds, respectively. Figure 3 displays the crosshair plot (sensitivity vs. false positive rates) for the three thresholds. Positive and negative LR from studies are presented in Fig. S1. Pooled DTA measures for each G6PD activity threshold are described (Table 2).

**Table 2:** Pooled DTA values for each threshold defined by Standard™ G6PD (SD Biosensor) test

G6PD activity threshold	Sensitivity (95%CI) & CoE	Specificity (95%CI) & CoE	+ LR (95%CI)	− LR (95%CI)
30%	99.1% (96.9%-99.7%) (4 studies, 3122 participants)	97.4% (95.2–98.4%) (4 studies, 3122 participants)	35.3 (20.7–60.5)	0.009 (0.003–0.031)
	High ⨁⨁⨁⨁	High ⨁⨁⨁⨁		
70%	95.7% (92.9–97.4%) (4 studies, 2371 participants)	92.8% (85.8–96.5%) (4 studies, 2371 participants)	13.2 (6.8–26.5)	0.046 (0.030–0.073)
	High ⨁⨁⨁⨁	High ⨁⨁⨁⨁		
80%	90.5% (78.4–96.2%) (4 studies, 2371 participants)	89.0% (76.9–95.1%) (4 studies, 2371 participants)	8.2 (4.1–16.0)	0.106 (0.049–0.227)
	Low ⨁⨁◯◯	Moderate ⨁⨁⨁◯		

*CI* confidence interval, *LR* likelihood ratio, + positive,*—*negative, *CoE* Certainty of the Evidence

Pooled results showed high DTA measures for all the thresholds being better for lower than the highest threshold. Positive and negative LR ranged from 8.2 to 35.3 and 0.009 to 0.106. The CoE for sensitivity and specificity was high for 30% and 70% thresholds, but for 80% threshold sensitivity was low and specificity was moderate due to concerns in the RoB, indirectness, inconsistency, and imprecision (See Table S3).

**Fig. 4:**
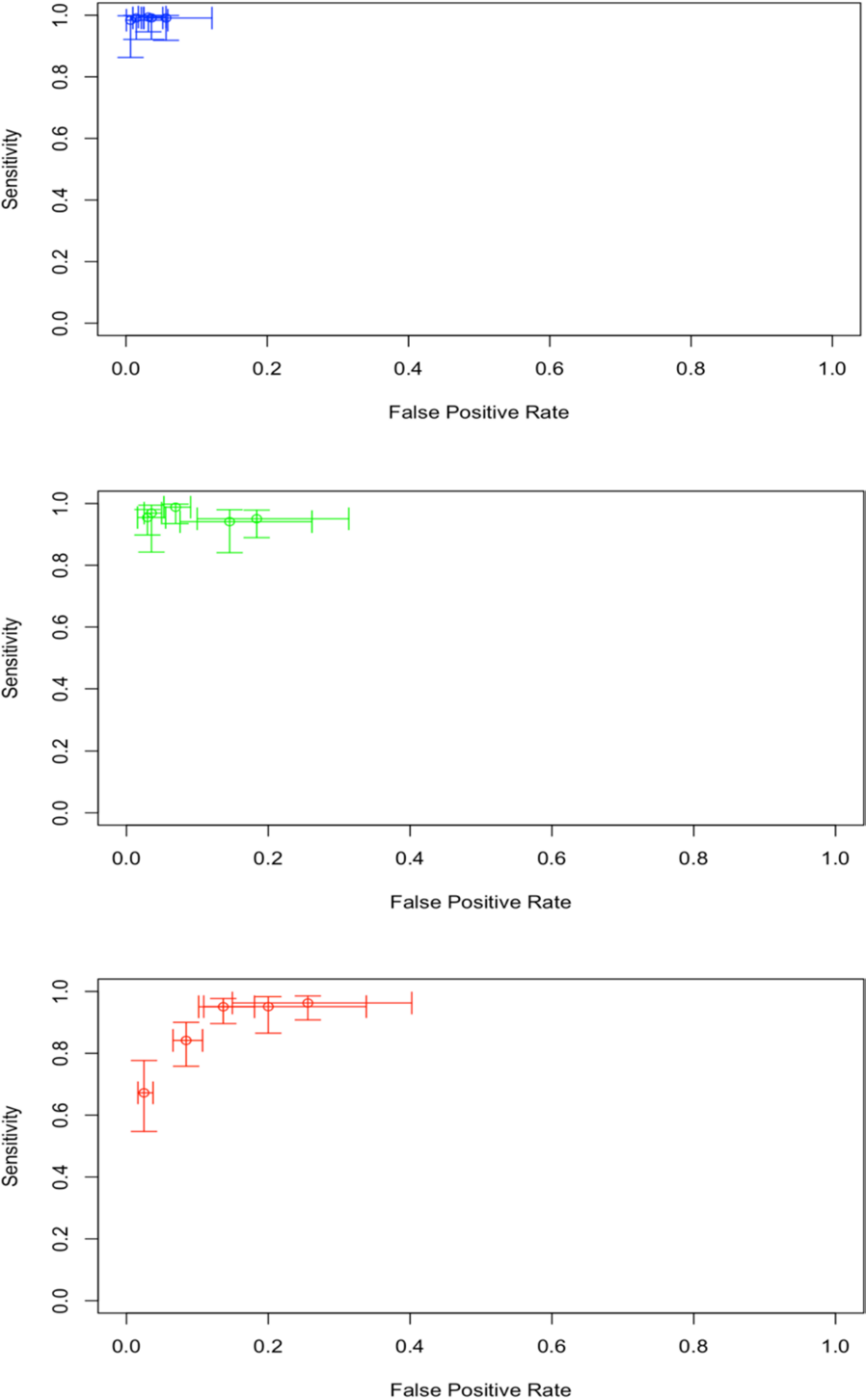
Crosshair plot for each threshold defined by the Standard^™^ G6PD (SD Biosensor) test

**Fig. 5:**
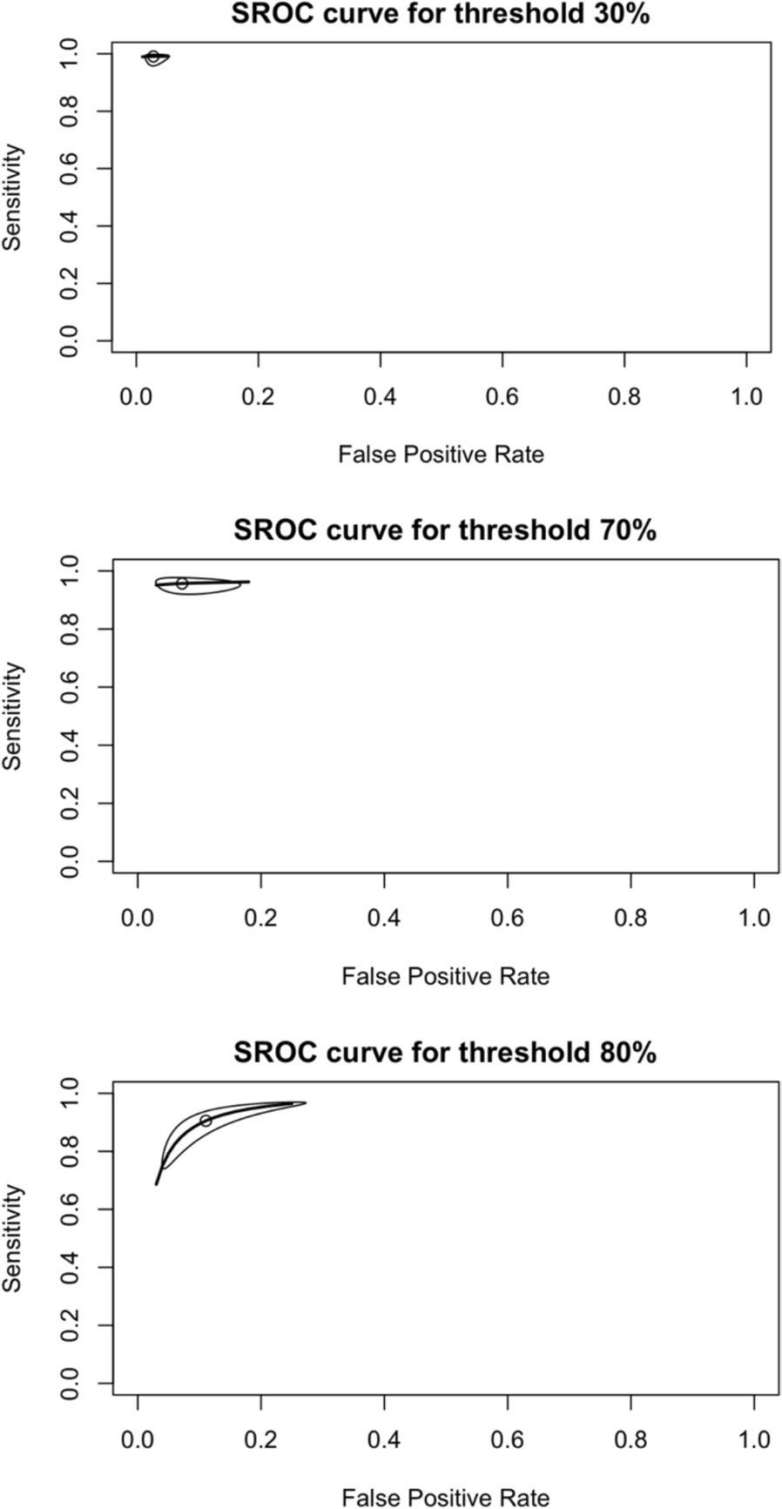
SROC curve (bivariate model) for different Standard G6PD (SD Biosensor) thresholds in venous blood samples

Low, moderate, and high heterogeneity were found in the results for the 30%, 70%, and 80% thresholds, respectively (Figs. 4 and 5**)**.

## Discussion

In this systematic review, four studies were included, evaluating 7,864 patients and found that StandG6PD-BS is a semi-quantitative PoC test with high sensitivity and specificity values to detect G6PDd at the different G6PD activity thresholds recommended by the manufacturer (30%, 70%, and 80%). The test showed better performance and CoE for 30% and 70%, compared with the 80% threshold. Likewise, the heterogeneity in the results was low, moderate, and high at 30%, 70%, and 80% thresholds, respectively.

The included studies were conducted in both high and low-middle-income countries, and participants were predominantly healthy adults with or without known G6PD status. This allows the decision-makers to assess the potential impact of implementing the StandG6PD-BS PoC test in the general population, particularly for uncomplicated malaria by *P. vivax*. The evidence suggests that introducing this test in tropical and subtropical malaria-endemic countries with *P. vivax* high transmission regions would be both feasible and desirable and could provide numerous benefits [5, 7, 27]. For instance, a recent study in Brazil [27] found that combining TQ treatment with the StandG6PD-BS test improved the response to radical cure treatment by enhancing adherence, reducing relapses, and increasing protection against new *P. vivax* infections.

The literature describes factors related to lower test accuracy that may not be explained just by the PoC test performance. A recent controlled study suggested that the StandG6PD-BS test performed well in venous blood, exhibiting good repeatability and inter-laboratory reproducibility [28]. However, the same study found that the reliability of the test was poor in discriminating between intermediate and low G6PD activities in lyophilized samples [28], emphasizing the need for further research in field-based scenarios. This study found similar estimates for sensitivity and specificity as reported by Addisu et al*.* [15]. However, this work is the first to conduct a complete systematic review approach through a comprehensive search and selection, a random model meta-analysis, risk of bias assessment, and certainty of evidence using the GRADE approach and following the methods recommended by the Cochrane collaboration.

In our approach, only results of venous blood samples were pooled because other sources, such as capillary and lyophilized blood, provided limited results due to small sample sizes in the studies and differences in the specimen collection and lyophilization methods. Nevertheless, some evidence suggests that capillary and lyophilized blood samples could drive less accuracy in the PoC test results [23, 28–30].

In the included studies, only one [26] used frozen venous blood to run the StandG6PD-BS test showing slightly less DTA than the fresh venous blood samples [26]. This could be due to the small number of participants, the specimen collection, or the storage method. An alternative cause is the study population (Thailand), which carries the G6PDd Mahidol phenotype with moderate enzymatic activity (30–70%). However, the StandG6PD-BS sensitivities and specificities at those levels are still around 90% [23, 28, 31].

The current evidence strongly supports the implementation of the PoC test for G6PD activity. At the individual level, it will enable the safe treatment of more patients with deficient and intermediate G6PD activity, diminishing the risk of recurrent malaria and acute haemolytic anaemia [5, 7, 32, 33]. At the healthcare systems level, it could reduce the associated costs and the burden on transfusion services by reducing the number of haemolytic crises caused by PQ or TQ in individuals with unknown G6PD status and malaria treatment [5, 7, 32, 34]. Additionally, this could impact parasite transmission rates when combined with other interventions [5, 7, 32]. Given that up to 50% of the patients with *P. vivax* malaria may experience relapses [5, 7], administering radical cure with PQ or TQ is essential for stopping morbidity-related and community transmission [5, 7, 27, 33].

This work has several strengths. This systematic review with meta-analysis is the first to evaluate the DTA of the StandG6PD-BS test. Moreover, state-of-the-art methodologies for conducting DTA studies was followed, as recommended by the Cochrane Handbook for Systematic Reviews of Diagnostic Test Accuracy [16]. The final report of this review following the recommendations by the PRISMA-DTA statement was prepared [35], with information on the pooled sensitivity and specificity for each threshold, which can facilitate decision-making in different clinical scenarios.

There are some limitations. First, the number of studies was low, preventing from performing additional analyses, such as sensitivity and subgroup analysis, so further studies should evaluate the diagnostic accuracy of this test in women, and participants of African ethnicities. Additionally, the quality of some studies was not optimal, although this analysis is robust enough to show adequate pooled DTA measures for venous blood samples. Future studies using capillary and lyophilized blood, along with field-based studies, are needed to determine the appropriate usage of this test. A study on barriers and facilitators for G6PD test implementation [36] identified three main barriers: perceived low risk of haemolysis, wrong perception of *P. vivax* malaria as a benign condition, and the cost of routine testing as part of the healthcare attention of malaria patients. A study conducted in Brazil on the operational challenges associated with pragmatic G6PD testing [37] found that the StandG6PD-BS PoC test was well accepted by both healthcare professionals and patients and can be performed at malaria treatment units in the Brazilian Amazon to inform treatment decisions with PQ. However, the authors found limitations linked to technical and cultural aspects that should be addressed when expanding screening to larger areas [37]. A recent scoping review [38] addressed the issue and found barriers regarding acceptability, such as time and logistics to travel to a health centre and concerns regarding the test steps compared to a qualitative test; feasibility, e.g., the use of the kit, concerns regarding the operative use and maintenance of the machine; and the value of the outcome, i.e., not knowing why the test was done.

## Conclusion

StandG6PD-BS is a PoC test with high sensitivity and specificity to detect G6PDd at the different thresholds recommended by the manufacturer (30%, 70%, and 80%). Implementing this kind of test in malaria-endemic areas can lead to early diagnosis of G6PDd, help to prevent haemolytic episodes triggered by PQ or TQ, and potentially impact malaria transmission.

## Supplementary Information

Additional file 1.

Additional file 2.

## Data Availability

The data are available on request from the authors.

## Abbreviations

CoE: Certainty of the evidence
DTA: Diagnostic test accuracy
FN: False negatives
FP: False positives
G6PD: Glucose-6-phosphate dehydrogenase
G6PDd: Glucose-6-phosphate dehydrogenase deficiency
GRADE: Grading of Recommendations Assessment, Development and Evaluation
PoC: Point-of-care
PQ: Primaquine
PRISMA-DTA: Preferred Reporting Items for Systematic Reviews and Meta-analyses of DTA Studies
QUADAS-2: Quality Assessment of Diagnostic Accuracy Studies 2
RoB: Risk of bias
ROC: Receiver operating characteristic
SoF: Summary of findings
TN: True negatives
TP: True positives
TQ: Tafenoquine
WHO: World Health Organization
