# Supplementary material for: Diagnostic accuracy of the point-of-care standard G6PD test™ (SD Biosensor) for glucose-6-phosphate dehydrogenase deficiency: a systematic review and meta-analysis
Source: Malar J. 2024 Nov 2;23:327. doi: 10.1186/s12936-024-05144-1 (PMC11531698; doi:10.1186/s12936-024-05144-1)
Supplement: Supplementary file 1 — Additional file 1. [file 12936_2024_5144_MOESM1_ESM.docx]

**Question**: Should STANDARD G6PD (TM) test manufactured by SD biosensor be used to diagnose G6PDd at 30% threshold in persons with suspicion?

| \| Sensitivity \| 0.99 (95% CI: 0.97 to 1.00) \| \| --- \| --- \| \| Specificity \| 0.97 (95% CI: 0.95 to 0.98) \| |  | \| Prevalences \| 7.1% \| 6.7% \| 7.4% \| \| --- \| --- \| --- \| --- \| |  |
| --- | --- | --- | --- | --- | --- | --- | --- | --- | --- | --- | --- |

| Outcome | № of studies (№ of patients) | Study design | Factors that may decrease certainty of evidence | | | | | Effect per 1,000 patients tested | | | Test accuracy CoE |
| --- | --- | --- | --- | --- | --- | --- | --- | --- | --- | --- | --- |
|  |  |  | Risk of bias | Indirectness | Inconsistency | Imprecision | Publication bias | pre-test probability of7.1% | pre-test probability of6.7% | pre-test probability of7.4% |  |
| **True positives** (patients with G6PDd at 30% threshold) | 4 studies 3122 patients | cross-sectional (cohort type accuracy study) | serious^a^ | not serious | not serious | not serious | strong association all plausible residual confounding would reduce the demonstrated effect dose response gradient | 70 (69 to 71) | 66 (65 to 67) | 73 (72 to 74) | ⨁⨁⨁⨁ High |
| **False negatives** (patients incorrectly classified as not having G6PDd at 30% threshold) |  |  |  |  |  |  |  | 1 (0 to 2) | 1 (0 to 2) | 1 (0 to 2) |  |
| **True negatives** (patients without G6PDd at 30% threshold) | 4 studies 3122 patients | cross-sectional (cohort type accuracy study) | serious^a^ | not serious | not serious | not serious | strong association all plausible residual confounding would reduce the demonstrated effect dose response gradient | 903 (884 to 914) | 907 (888 to 918) | 900 (882 to 911) | ⨁⨁⨁⨁ High |
| **False positives** (patients incorrectly classified as having G6PDd at 30% threshold) |  |  |  |  |  |  |  | 26 (15 to 45) | 26 (15 to 45) | 26 (15 to 44) |  |

#### Explanations

a. Two studies graded as with high risk of bias

**Question**: Should STANDARD G6PD (TM) test manufactured by SD biosensor be used to diagnose G6PDd at 70% threshold in persons with suspicion?

| \| Sensitivity \| 0.96 (95% CI: 0.93 to 0.97) \| \| --- \| --- \| \| Specificity \| 0.93 (95% CI: 0.86 to 0.96) \| |  | \| Prevalences \| 7.1% \| 6.7% \| 7.4% \| \| --- \| --- \| --- \| --- \| |  |
| --- | --- | --- | --- | --- | --- | --- | --- | --- | --- | --- | --- |

| Outcome | № of studies (№ of patients) | Study design | Factors that may decrease certainty of evidence | | | | | Effect per 1,000 patients tested | | | Test accuracy CoE |
| --- | --- | --- | --- | --- | --- | --- | --- | --- | --- | --- | --- |
|  |  |  | Risk of bias | Indirectness | Inconsistency | Imprecision | Publication bias | pre-test probability of7.1% | pre-test probability of6.7% | pre-test probability of7.4% |  |
| **True positives** (patients with G6PDd at 70% threshold) | 4 studies 2371 patients | cross-sectional (cohort type accuracy study) | serious^a^ | serious | not serious | not serious | strong association all plausible residual confounding would reduce the demonstrated effect dose response gradient | 68 (66 to 69) | 64 (62 to 65) | 71 (69 to 72) | ⨁⨁⨁⨁ High |
| **False negatives** (patients incorrectly classified as not having G6PDd at 70% threshold) |  |  |  |  |  |  |  | 3 (2 to 5) | 3 (2 to 5) | 3 (2 to 5) |  |
| **True negatives** (patients without G6PDd at 70% threshold) | 4 studies 2371 patients | cross-sectional (cohort type accuracy study) | serious^a^ | serious | serious^b^ | not serious | strong association all plausible residual confounding would reduce the demonstrated effect dose response gradient | 862 (797 to 896) | 866 (801 to 900) | 859 (795 to 894) | ⨁⨁⨁⨁ High |
| **False positives** (patients incorrectly classified as having G6PDd at 70% threshold) |  |  |  |  |  |  |  | 67 (33 to 132) | 67 (33 to 132) | 67 (32 to 131) |  |

#### Explanations

a. Two studies graded as with high risk of bias

b. Two studies with non-overlapping CI and with non-overlapping points in crosshair graphs.

**Question**: Should STANDARD G6PD (TM) test manufactured by SD biosensor be used to diagnose G6PDd at 80% threshold in persons with suspicion?

| \| Sensitivity \| 0.91 (95% CI: 0.78 to 0.96) \| \| --- \| --- \| \| Specificity \| 0.89 (95% CI: 0.77 to 0.95) \| |  | \| Prevalences \| 7.1% \| 6.7% \| 7.4% \| \| --- \| --- \| --- \| --- \| |  |
| --- | --- | --- | --- | --- | --- | --- | --- | --- | --- | --- | --- |

| Outcome | № of studies (№ of patients) | Study design | Factors that may decrease certainty of evidence | | | | | Effect per 1,000 patients tested | | | Test accuracy CoE |
| --- | --- | --- | --- | --- | --- | --- | --- | --- | --- | --- | --- |
|  |  |  | Risk of bias | Indirectness | Inconsistency | Imprecision | Publication bias | pre-test probability of7.1% | pre-test probability of6.7% | pre-test probability of7.4% |  |
| **True positives** (patients with G6PDd at 80% threshold) | 4 studies 2371 patients | cross-sectional (cohort type accuracy study) | serious^a^ | serious | serious^b^ | serious^c^ | all plausible residual confounding would reduce the demonstrated effect dose response gradient | 64 (56 to 68) | 61 (53 to 64) | 67 (58 to 71) | ⨁⨁◯◯ Low |
| **False negatives** (patients incorrectly classified as not having G6PDd at 80% threshold) |  |  |  |  |  |  |  | 7 (3 to 15) | 6 (3 to 14) | 7 (3 to 16) |  |
| **True negatives** (patients without G6PDd at 80% threshold) | 4 studies 2371 patients | cross-sectional (cohort type accuracy study) | serious^a^ | serious | serious^b^ | serious^c^ | strong association all plausible residual confounding would reduce the demonstrated effect dose response gradient | 827 (714 to 883) | 830 (717 to 887) | 824 (712 to 881) | ⨁⨁⨁◯ Moderate |
| **False positives** (patients incorrectly classified as having G6PDd at 80% threshold) |  |  |  |  |  |  |  | 102 (46 to 215) | 103 (46 to 216) | 102 (45 to 214) |  |

#### Explanations

a. Two studies assessed as with high risk of bias.

b. High heterogeneity detected in crosshair graphs.

c. Wide CI
